# A Proposed Mechanism for the Interaction of the Segmentation Clock and the Determination Front in Somitogenesis

**DOI:** 10.1371/journal.pone.0001561

**Published:** 2008-02-06

**Authors:** Moisés Santillán, Michael C. Mackey

**Affiliations:** 1 Campus Monterrey, Centro de Investigación y Estudios Avanzados del Instituto Politécnico Nacional (IPN), Apodaca, Nuevo León, México; 2 Centre for Nonlinear Dynamics, McGill University, Montreal, Québec, Canada; Katholieke Universiteit Leuven, Belgium

## Abstract

**Background:**

Recent discoveries in the field of somitogenesis have confirmed, for the most part, the feasibility of the clock and wavefront model. There are good candidates for both the clock (various genes expressed cyclically in the tail bud of vertebrate embryos have been discovered) and the wavefront (there are at least three different substances, whose expression levels vary along the presomitic mesoderm [PSM], that have important effects on the formation of somites). Nevertheless, the mechanisms through which the wavefront interacts with the clock to arrest the oscillations and induce somite formation have not yet been fully elucidated.

**Principal Findings:**

In this work, we propose a gene regulatory network which is consistent with all known experimental facts in embryonic mice, and whose dynamic behaviour provides a potential explanation for the periodic aggregation of PSM cells into blocks: the first step leading to the formation of somites.

**Significance:**

To our knowledge, this is the first proposed mechanism that fully explains how a block of PSM cells can stop oscillating simultaneously, and how this process is repeated periodically, via the interaction of the segmentation clock and the determination front.

## Introduction

Segmentation of the body axis is a basic characteristic of many animal species ranging from invertebrates to mammals. The vertebrate body is organized, along the antero-posterior (AP) axis, in a series of functionally equivalent units, each comprising a vertebra, its associated muscles, peripheral nerves, and blood vessels. These units originate from the earlier pattern of the embryonic somites, which are blocks of cells generated in a rhythmic fashion from the mesenchymal presomitic mesoderm (PSM).

Several models of somitogenesis have been put forward. However, the *clock and wavefront* model of Cooke and Zeeman [1] has found the widest acceptance and applicability. According to this model, reviewed in [2], the segmental pattern is established in the PSM by a mechanism involving an oscillator (the segmentation clock), which is hypothesized to set the periodicity of the process, and a travelling wavefront of cell change that sweeps anteriorly to posteriorly through the PSM, arresting the oscillation, and inducing (or permitting) somite maturation.

The discovery in 1997 of an oscillatory expression of the gene *c-hairy1* in the PSM of chick embryos provided the first clear molecular evidence for a segmentation clock [3]. Since, several other cycling genes have been identified in various vertebrate species (mouse, chicken, fish and frog). While all of the cycling genes initially characterized were found to be targets or components of a single signalling pathway (the Notch signalling pathway), a second group of cyclic genes related to the Wnt signalling pathway was recently discovered in mice [4]. The available experimental evidence suggests that the Wnt and Notch signalling pathways interact and influence each other in the segmentation clock.

In 2001 Dubrulle et al. [5] described a graded expression of Fibroblast Growth Factor (FGF) signalling within the PSM. More recently, a second pathway was found to establish a signalling gradient within the PSM: the Wnt-pathway [4]. While travelling along the AP axis, a cell would experience decreasing levels of FGF and Wnt signalling until the levels drop below a critical threshold. This region has been termed the determination front, and it is believed to mark a region of developmental change. It is thought that the interaction between the segmentation clock and these gradients at the level of the determination front defines the segment size. Furthermore, a third signalling gradient in the PSM has been identified [6]. The retinoic acid (RA) pathway is graded in the opposite direction relative to FGF signalling and counteracts the latter, thus influencing the position of the determination front.

It is generally proposed that the interaction between the segmentation clock and the gradient of signalling pathways specifies a segment in the anterior PSM. A crucial question in this scenario, however, is: “How is this interaction achieved?”

Aulehla and Herrmann [7] proposed that Wnt signalling in the PSM of mice activates oscillations of the Wnt cyclic genes, which then are linked to oscillations of Notch cyclic genes. Since Wnt signalling is graded in the PSM, the signal to activate the oscillations would eventually drop below a threshold and therefore, the oscillations in the anterior PSM would stop. According to Aulehla and Herrmann, this would define the boundary between cells that are still capable of oscillation and cells in which the oscillations have been arrested. Nevertheless, using a mathematical modelling approach Rodríguez-González et al. [8] demonstrated that, although this mechanism is capable of explaining cycle arrest, it is unable to predict the formation of well-defined PSM segments.

In a recent paper, Goldbeter et al. [9] demonstrated that the antagonistic gradients of RA and FGF along the presomitic mesoderm might lead to the coexistence of two stable steady states. They further proposed that this bistability is associated with abrupt switches in the levels of FGF and RA signalling, which permit the synchronized activation of segmentation genes in successive cohorts of PSM cells in response to the segmentation clock, thereby defining the future segments. However, they did not provide a mechanism to explain how interactions between the segmentation clock and the switching of FGF and RA levels might take place.

Thus, the mechanisms by which the segmentation clock interacts with the gradient of signalling pathways in the PSM remain a mystery, despite great advances in the last few years. Some recent papers have suggested models to explain how the somite clock can be stoped by the FGF or Wnt gradients [8], [10]. However, none of them accounts for the simultaneous arrest of cycling in all the cells that will eventually form a somite.

In this paper we approach this problem from a mathematical modelling perspective. Based upon extant experimental evidence on mice embryos, we propose a regulatory network involving two genes in the Notch and FGF/Wnt signalling pathways and show that its dynamic behaviour is sufficient to explain the rhythmic segmentation of PSM cells in this species.

## Methods

### Model development

Several modelling studies support the claim that, given the involved regulatory mechanisms, the expression of one or more genes under the Notch regulatory pathway can oscillate spontaneously. They also suggest that the underlying mechanism is a simple negative feedback loop with relatively long time delays due to transcription and translation of the corresponding genes [11]–[14].

However, the experimental evidence regarding the interaction between the Notch and FGF/Wnt signalling pathways is still scarce. Recently, however, experimental data from the analysis of the regulation of Delta-like1 (*Dll1*), a Notch ligand strongly expressed in the PSM, indicate that *Dll1* in the paraxial mesoderm and tail bud of mouse embryos is regulated by Wnt signalling [15], [16]. Additionally, naked cuticle, a negative regulator of Wnt signalling, has been identified as a cyclic gene under the control of Notch signalling [17]. These examples indicate a close interaction between these two pathways. However, the precise mechanisms of cross talk have not yet been elucidated. On the other hand Aulehla et al. [4] found that the expression of gene *Axin2* (which is up-regulated by Wnt) oscillates in mice embryos. They further proposed that these oscillations are originated by the following negative feedback loop: protein Axin2 binds protein Dvl —dishevelled, dsh homolog 1 (Drosophila)— and so decreases the concentration of this last protein free form, which is an activator of gene *Axin2*.

**Figure 1:**
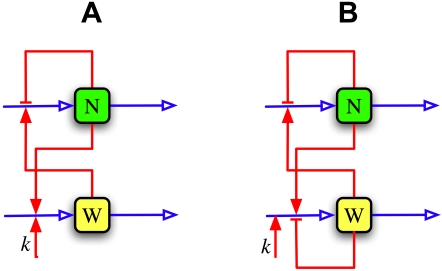
Schematic representations of the regulatory pathway involving genes under the Notch (N) and FGF/Wnt (W) regulatory pathways. The network nodes represent the enzyme levels, the blue arrows pointing into the nodes account for the processes of transcription and translation lumped together, and the blue arrow exiting from the nodes represent enzyme degradation.The regulatory processes are represented by red lines: the lines ending with arrowheads (bars) correspond to up-regulation (down-regulation). Finally, *k* accounts for the up-regulation of either gene *W* of the activity of its encoded protein by FGF and/or Wnt.

Based on the above considerations we propose two gene regulatory networks, one of which is a simplified version of the other. These networks are schematically represented in Figures 1A and 1B. Notice that both involve two genes, one under the Notch (N) and the other under the FGF/Wnt (W) signalling pathways. In both cases, gene N self inhibits its own expression (via a simple negative feedback loop) and up-regulates the expression of W; likewise, W up-regulates the expression of N. The mutual interaction of N and W gives rise to a double positive loop. We also assume that in both networks either the expression of gene W or the activity of protein *W* is up regulated by the FGF and/or Wnt levels (this is accounted for by *k* which is a monotone increasing growing function of the FGF and/or Wnt concentrations). Finally, the network of Figure 1B considers one further interaction in which gene W down-regulates its own expression.

The interactions described above for the network of Figure 1A can be translated into a mathematical model as follows:
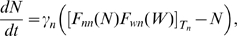
(1)

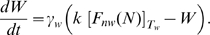
(2)In these equations, *N* and *W* are respectively proportional to the active protein levels resulting from the expression of genes N and W. The model equations have been normalized so that *N* and *W* are dimensionless and their maximum possible value is one, which they attain when the corresponding genes are maximally expressed. The degradation rates for proteins *N* and *W* are *γ_n_* and *γ_w_*, respectively. *T_n_* and *T_w_* are the total time delays due to transcription, mRNA processing, and translation of both genes, and the notation [L]*_T_* indicates that all variables inside the square brackets are delayed a time *T*, e.g. [*x*(*t*)]*_T_* = *x*(*t−T*). *F_nn_*, *F_wn_*, and *F_nw_* are functions representing the network regulatory interactions: *F_nn_*(*N*) accounts for the self inhibition of gene N, and so it must be a decreasing function; *F_wn_*(*W*) and *F_nw_*(*N*) respectively stand for the influence that proteins *W* and *N* have on the expression of genes N and W. Since these two interactions up-regulate the targeted genes, both functions are monotone increasing functions of their argument. Finally, as discussed above, *k* is an increasing function of the FGF and/or Wnt levels, and accounts for the assumed positive influence of these chemical species on either the expression level of gene W or the activity of the corresponding protein.

Monotone decreasing and increasing Hill-type equations as follows can model the regulatory functions:
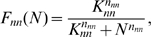


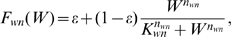


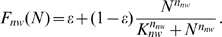
The factor *ε*<1 represents transcriptional leakage.

To model the network of Figure 1B, Equation (2) has to be modified to take into account the self-regulation of gene W as follows:
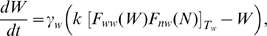
(3)where the function *F_ww_*(*W*) accounts for the self-inhibition of gene W and is defined by
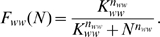



### Parameter estimation

Monk [14], Giudicelli and Lewis [18], Hirata et al. [12], and Bernard et al. [11] report half-life times of about 25 min (which corresponds to a degradation rate around 0.03 min^−1^) for different proteins of the Hes family and their corresponding mRNA. Since the corresponding genes are in the Notch signalling pathway we take
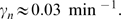
We were unable to find equivalent data for genes under the Wnt signalling pathway and so we assume that
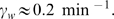



Rodríguez-González et al. [8] estimated the time delays due to transcription, mRNA processing, and translation for various genes in the Notch and Wnt signalling pathways in mice. For genes *Hes1* and *Lfng* (under the Notch pathway) they respectively estimated the following ranges: 11 to 33 min and 16 to 66 min. Meanwhile, for gene *Axin2* (under the Wnt pathway) the estimated range is 45 to 116 min. Here we choose
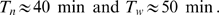



For the regulatory functions we take the following parameter values:
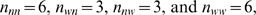
and
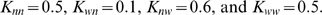



Finally, we chose the transcriptional leakage parameter to be
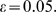



Given that many of the parameters were not estimated from reported experimental data, it is necessary to assess the robustness of the model results to variations in the parameter values.

### Numerical methods

The models' time-delay differential equations were numerically solved using the software xppaut. The same program was used to calculate the corresponding bifurcation diagrams.

## Results

### *N* and *W* oscillations

**Figure 2:**
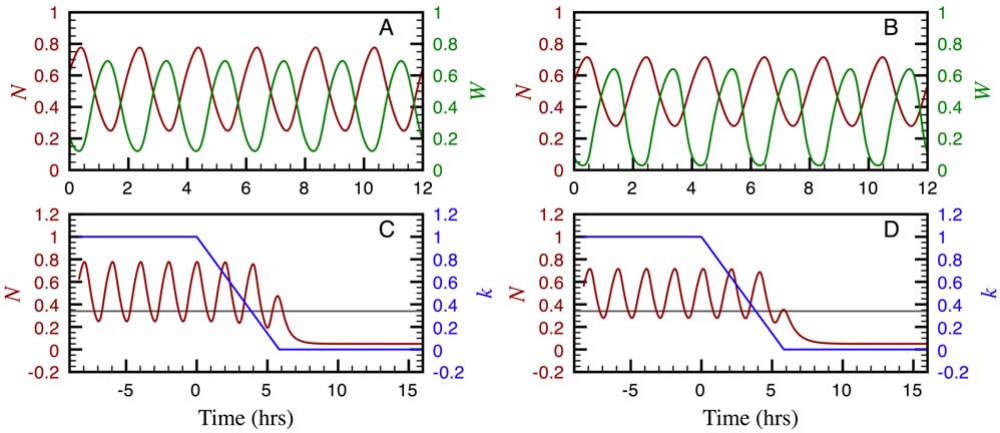
A and B, plots of *N vs. t* and *W vs. t* resulting from the numerical solution, with *k* = 1, of the equations corresponding to the networks in Figures 1A and 1B, respectively. C and D, plots of *N vs. t* and *k vs. t* as *k* decays, for the models corresponding to the networks in Figures 1A and 1B. The time at which the cell leaves the TB and *k* starts decreasing is *t* = 0. The horizontal grey lines represent the *k*′ bifurcation value below which the oscillations are arrested. All other parameters were set to the values estimated in the section on parameter estimation.

It is known that various genes under the Notch and Wnt signalling pathways oscillate in cells located within the tail bud (TB), where high FGF and Wnt levels are found. Since we assume in our models that either the gene W is up-regulated or the corresponding protein is activated by FGF and/or Wnt, and that this interaction is accounted for by the function *k*≤1, the TB conditions can be simulated by setting *k* = 1. After doing so, we numerically solved the equations corresponding to both models and plotted the results in Figures 2A and 2B. Notice that in both models *N* and *W* oscillate with a period of about 2 hrs, and that the *W* oscillations are out of phase by half a cycle with respect to those of *N*. These two observations are in agreement with reported experimental results in [19]. However, they are not independent of the parameter values. In the next paragraph we address this issue.

After carefully inspecting how variations on all the parameter values influence the oscillatory behaviour of the network in Figure 1A we found that:

- Given the degradation rate *γ_n_* for *N*, the Hill coefficient of the negative feedback regulatory function (*F_nn_*) must satisfy *n_nn_*≥5 in order for the system to show sustained oscillations. Larger values of *γ_n_* would allow the existence of a stable limit cycle (stationary oscillations) with smaller *n_nn_* values.
- The oscillation period is essentially determined by the parameters *γ_n_* and *T_n_*: the longer the time delay, *T_n_*, and the smaller the degradation rate, *γ_n_*, the longer the oscillation period. Given that we consider *γ_n_* fixed (it is one of the few parameters that we were able to estimate from experimental data), we picked the time delay necessary to have a 2 hrs cycling period. Nonetheless, it is important to emphasize that the resulting time delay value lies within the range estimated in the section on parameter estimation.
- The phase shift between the *N* and *W* oscillations depends on the difference between the time delays *T_n_* and *T_w_*. For the *N* and *W* curves to be out of phase by half a cycle, *T_w_* must obey either of the following relations: 40 min≤*T_w_*≤60 min or 150 min≤*T_w_*≤170 min, given *T_n_*≈40 min.
- Finally, the larger the *W* degradation rate, *γ_w_*, the larger the corresponding oscillation amplitude. Here, we chose a *γ_w_* value such that the amplitudes of the *W* and *N* oscillations are comparable.

We also analysed the influence that changes on the parameters of the network in Figure 1B have on the system oscillatory behaviour. From this analysis we observed that:

- In this network there are two subsystems capable of generating sustained oscillations: the delayed negative feedback loop corresponding to the self-inhibition of gene N, and that corresponding to the self-inhibition of gene W. Given the degradation rate *γ_n_*, the Hill coefficient *n_nn_* must be larger than 5 in order for the corresponding subsystem to oscillate spontaneously. Similarly, given the value of *γ_w_*, *n_ww_*≥5 in order for the negative feedback loop associated to gene W to show a cyclic behaviour.
- If the parameters of the N and W negative feedback loops are set such that the N subsystem can, and the W cannot, oscillate spontaneously, the N subsystem can make the W subsystem oscillate whenever *K_nw_*<0.8 and *K_wn_*<0.5. Similarly, given the network symmetry, the W subsystem can cause the N subsystem to oscillate if *K_nw_*<0.5 and *K_wn_*<0.8.
- The oscillation frequency of the N and W subsystems (when they can oscillate spontaneously) largely depends on their respective degradation rates and time delays: the larger the time delay and the smaller the degradation rate, the longer the corresponding oscillation period.
- When both subsystems can oscillate by themselves and they are coupled as in Figure 1B, they synchronize even if their natural cycling frequencies differ by less than 10%, approximately. In this case, the phase shift between the *N* and *W* oscillations depends on the difference between the time delays *T_n_* and *T_w_*. For the *N* and *W* curves to be out of phase by half a cycle, *T_w_* must obey either of the following relations: 40 min≤*T_w_*≤60 min or 150 min≤*T_w_*≤170 min, given *T_n_*≈40 min.

### Bifurcation diagrams

**Figure 3:**
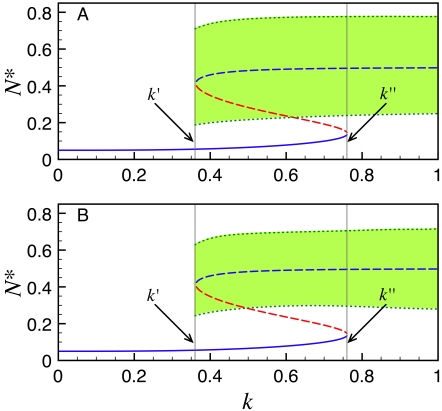
Bifurcation diagrams predicting the stationary behaviour of the models corresponding to the networks in Figures 1A (A) and 1B (B). The solid blue line denotes the *N* ^*^ values in a stable steady state, as a function of *k*; the dashed red line represents a saddle node; the dashed blue line indicates an unstable steady state inside a limit cycle; and the green dotted lines correspond to the upper and lower limits of the *N* ^*^ oscillations in a stable limit cycle. All other parameters were set to the values estimated in the section on parameter estimation.

As the embryo grows the tail bud recedes and, while doing so, leaves some cells behind. After a cell leaves the TB, the FGF and Wnt levels in the surrounding medium start decreasing until they reach a given threshold and, according to the clock and wavefront model, arrest clock oscillations and promote somite formation. In our two proposed networks, decreasing *k* simulates a decrease on the FGF/Wnt levels. To better understand the system stationary behaviour as a function of *k*, we calculated the bifurcation diagrams for the models corresponding to the networks in Figures 1A and 1B and plotted the results in Figures 3A and 3B, respectively.

In the diagram of Figure 3A we observe that for values of *k* larger than *k*″ = 0.76, only a stable limit cycle (stationary oscillatory state) exists; for *k*<*k*′ = 0.36, only a stable steady state —corresponding to a very low induction level of gene N— is found; and for *k*′<*k*<*k*″ the limit cycle and the steady state coexist. In this case, the limit cycle originates from the time-delayed negative feedback loop involved in the self-regulation of gene N, while the bistable behaviour is a consequence of the reciprocal up-regulation of genes N and W. The parameter-value constraints necessary to obtain this diagram are:

- The Hill coefficients, *n_wn_* and *n_nw_*, of the *F_wn_* and *F_nw_* functions must be larger than 2 for the model to show bistability.
- Parameters *K_wn_* and *K_nw_* can take a wide range of values without affecting the bifurcation diagram appearance and only modifying the values of *k*′ and *k*″. The must important restriction for them is that *K_nw_*>*K_wn_*, because otherwise the oscillations damp out for *k*>*k*′.

The bifurcation diagram in Figure 3B is quite similar to that in Figure 3A, the most noticeable difference being that the oscillation amplitude is smaller. The restrictions the parameter values have to satisfy to obtain a diagram like this are the same. In this case, bistability also originates from the reciprocal up-regulation of genes N and W, as in the circuit shown in Figure 3A. Finally, the delayed negative feddback loop of either gene N or gene W, or both, can be tuned to oscillate spontaneously and in all cases a behaviour similar to the one predicted by this diagram will be obtained.

From these bifurcation diagrams we can see that the two proposed models predict that once a PSM cell leaves the TB —and so the FGF/Wnt levels start decreasing— it will keep oscillating with a more or less constant amplitude until *k* reaches the value *k*′. After that, the oscillations will abruptly stop and the system will jump to the low N-induction-level stable steady state. This behaviour is consistent with the hypothesis of Aulehla and Pourquié [19] that a decrease on the Wnt level can make oscillations stop, as well as with the experimental observations (reviewed in [19]) reporting that the Wnt pathway is necessary for a proper functioning of the segmentation clock. On the other hand, Aulehla et al. [4] and Hirata et al. [12] observed oscillations of Wnt pathway activity in the absence of Notch signalling or Notch pathway oscillations. This behaviour can clearly not been reproduced by the network in Figure 1A, since the only source of oscillations there is the delayed negative feedback loop of gene N. However, from the results discussed above, the network in Figura 1B can mimmic this observation if the parameters of the delayed negative feedback loop of gene W are set such that this subsystem oscillates spontaneously.

### Interaction of the segmentation clock and the formation front

As noted above, the FGF and Wnt levels start decreasing right after a cell leaves (or is left behind by) the PSM, and continue to do so until they eventually reach a threshold value that triggers cycle arrest. In Figures 2C and 2D we show how, according to the two proposed models, a PSM cell responds to a *k* value linearly decreasing in time, after remaining constant for a few hours. Note that in both cases the system still undergoes a few damped oscillations after *k* drops bellow the bifurcation value, *k*′. The time delays in the gene regulatory pathway are the reason why the system does not stop cycling immediately at the bifurcation point, as one would expect from the bifurcation diagram of Figures 3A and 3B.

We used the model corresponding to the network in Figure 1B to simulate the time evolution of 18 cells distributed along the PSM, with a separation of 20 min between consecutive cells. If the tail bud recedes at constant velocity and keeps leaving cell behind in a steady fashion, the distance between two PSM cells is proportional to the difference of elapsed times since their leaving the TB. Given that the oscillation period is 2 hrs, the considered cell set spans a PSM region three periods long. To account for the cell separation, we simply assumed in our simulations that the function describing how parameter *k* decays in time is delayed in proportion to how much later a given cell left the TB. In other words, if *k*(*t*) describes the time evolution of parameter *k* for the first cell, *k*(*t*-(*i*-1)Δ*T*) is the corresponding function for the *i*-th cell, with Δ*T* = 20 min.

**Figure 4:**
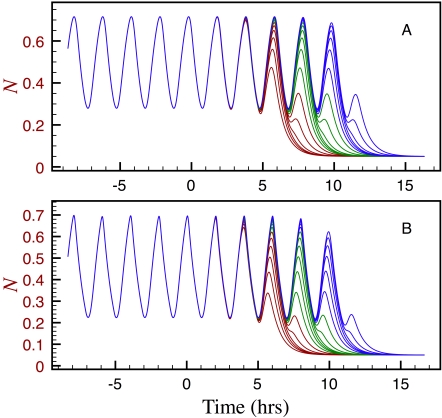
Plots of *N vs. t* representing the time-evolution of 18 cells distributed along the PSM, with a separation of 20 min between adjacent cells, with *k* evolving as in Figures 2C and 2D. Under the assumption that the tail bud recedes at constant velocity and keeps leaving cells behind in a steady fashion, the distance between two PSM cells is proportional to the time difference elapsed since leaving the TB. The simulations plotted in A were carried using the model given by Equations (1) and (3). For the simulations plotted in B we employed the model given by Equations (4) and (5), which include additional delays associated to the interactions between the Notch and FGF/Wnt pathways. In both cases, the curves corresponding to cells closer to the TB stop oscillating latter. For more clarity, the curves corresponding to the first, second and third sets of six cells are plotted in red, green, and blue, respectively. All the parameters were set to the values estimated in the section on parameter estimation.

In Figure 4A we show the results of the simulations described in the previous paragraph. The curves corresponding to the cells within the first, second and third periods are respectively coloured in red, green, and blue. Notice that all the cells within one period oscillate almost synchronously for a while, but their behaviour differs greatly during the last few cycles. This is a natural consequence of the fact that *k* reaches the bifurcation value *k*′ at different stages of each cell's cycle. On the other hand, the cells repeat the overall behaviour in the next period, in accordance with the cyclic nature of the phenomenon. Finally, it is important to emphasize that most of the cells within one period stop oscillating at very similar times. This sort of discrete behaviour may explain the clustering of PSM cells into somites once oscillations stop. These processes can be visualized in Movie S1, where each cell's time-evolution (as given by the *N vs. t* curves in Figure 4A) is animated.

To carry out the simulations plotted in Figure 4A, the parameters associated with the delayed negative feedback loops of genes N and W were set so that both subsystems can oscillate spontaneously. We repeated the simulations by assuming that only the W subsystem is able to generate sustained oscillations. Our results (not shown) in this case were similar to those reported in Figure 4A, except that the times at which the cells stop oscillating are almost uniformly distributed. From the discussion in the previous paragraph, these last simulations are unable to explain the segmentation of PSM cells into the blocks that will form somites.

We have assumed in our models that the interaction between the Wnt and Notch pathways is very rapid relative to the transcription, post-transcriptional modification, and translation processes. However, the genes N and W are most likely not directly connected, but affect each other through a series of intermediate steps that involve other chemical species. To account for this, we modified the model corresponding to the network in Figure 1B —Equations (1) and (3)— as follows:
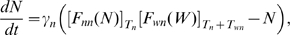
(4)

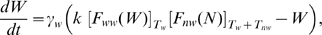
(5)where *T_wn_* and *T_nw_* represent additional time delays resulting from the non-instantaneous nature of the interactions between the Wnt and the Notch pathways. We repeated the simulations in Figure 4A using this modified model, for many values of *T_wn_* and *T_nw_*. The results for *T_wn_* = *T_nw_* = 10 min are plotted in Figure 4B and animated in Movie S2. Observe that the dynamic behaviour of the whole cell set is very similar to that of simulations in Figure 4A. We repeated the simulations with *T_wn_* = *T_nw_* = 20, 30, 40 min and noted that: (1) when the delays are equal to 20 min, many of the cells within one cycle still tend to stop oscillating at similar times, but not as markedly as in Figures 4A and 4B; (2) when the delays are equal to 30 min, the cells stop cycling at almost uniformly distributed times; and (3) when the delays are equal to 40 min, the oscillatory behaviour of the whole system is lost and replaced by a chaotic-like behaviour.

## Discussion

Recent discoveries in somitogenesis research have confirmed, for the most part, the basic veracity of the clock and wavefront model. We now know various genes that are expressed cyclically in the tail bud of vertebrate embryos, and at least three different substances whose expression levels vary along the PSM. Thus, there are good candidates for both the clock and the wavefront. Nevertheless, the mechanisms by which the wave front interacts with the clock to arrest the oscillations and induce somite formation have not yet been fully elucidated. In this paper we have proposed two gene regulatory networks (one being a simplified version of the other) consistent with the known experimental facts in mice, and whose dynamic behaviour provides a potential explanation for the periodic aggregation of PSM cells into blocks: the first step leading to the formation of somites.

In agreement with previous models [11]–[14], we assumed in both networks that a gene under the Notch regulatory pathway (N) inhibits its own expression, and that this regulation involves time delays due to transcription, mRNA processing, and translation. We further assumed —based on evidence in favour of a tight interaction between the Notch and Wnt signalling pathways in mice [19]— that this gene and another subject to regulation by FGF and/or Wnt (W) interact by up-regulating each other's expression. Finally, in the second proposed network we assumed that gene W is self-regulated via a delayed negative feedback loop, based on the experimental evidence reported in [4].

We paid special attention to the estimation of the parameters in the two proposed models from experimental results. Given the scarcity of available data, we were only able to find rough estimates for the degradation rates and time delays. We carried out numerical parameter sensitivity analysis for the rest of the parameters in the two models to remedy this deficiency. Regarding the time delays in the negative feedback loops, we estimated them by adding the times necessary for gene transcription, to eliminate introns from the resulting mRNA, to shuttle the processed mRNA into the cytoplasm, and to translate it. There exist other possible contributions to these delays, such as post-translational modification of some enzymes or enzyme-to-enzyme interactions. Nevertheless, we expect them to be at least one order of magnitude shorter than the estimated delays (which are of the order of 50 min), and this justifies our not considering them explicitly.

A careful analysis of the dynamic behaviour of the simplified network (Figure 1A) revealed that, with proper parameter values, the time-delayed negative feedback loop involved in the self regulation of gene N can give rise to sustained oscillations, while bistability can arise as a result of the reciprocal up-regulation of genes N and W. The oscillatory system behaviour matches the experimental results in which the oscillation period is about 2 hrs in mice, while *N* and *W* oscillate out of phase by half a cycle. We explored the parameter space to test the robustness of the model results, and concluded that the previously described behaviour can be obtained with a wide range of parameter values, with orders of magnitude compatible with the experimental data.

In the second network (Figure 1B), there are two subsystems capable of generating oscillations: the delayed negative feedback loops associated to genes N and W. From an exhaustive numerical study we found that each of these subsystems can make the other oscillate, via the proposed reciprocal up-regulation of genes N and W; and that they can synchronize if their parameters are set so that the two of them can oscillate spontaneously. In all the cases, we found wide parameter ranges for which the whole system oscillates with a period of about 2 hrs, while the *N* and *W* oscillations are out of phase by half a cycle. This model, in contrast to the simplified one, agrees with the results of Aulehla et al. [4] and Hirata et al. [12] who observed oscillations of Wnt pathway activity in the absence of Notch signalling or Notch pathway oscillations. Nonetheless, we believe that the analysis of the simplified network still is useful to understand the origin of some of the dynamic features of the present one.

It has been observed that the oscillation period of the somitogenesis clock gradually increases as the cells are displaced in the PSM [18]. Conversely, the present models predict that the oscillations continue with a more or less constant frequency until they stop, as seen in Figures 2C and 2D. Tiedemann et al. [10] studied a model in which oscillations are induced by a delayed negative feedback loop, as in our models, and concluded that the slowing down of oscillations can be explained by assuming that the mRNA degradation rate depends on the extracellular FGF concentration. This additional interaction cannot be introduced in our models in their current form because they lump together the corresponding mRNA and protein dynamics into a single equation. We have prefered having models as simple as possible to better understand their dynamics, rather than having models capable of reproducing all details of the system dynamics. However, given the similarity of our models with that of Tiedemann et al., we are certain that by increasing the model's dimensionality and introducing a mRNA degratation rate that is a growing function of *k*, we would be able to reproduce the observed increase on the oscillation period.

We further tested the response of a set of cells distributed along the PSM to a decay of the FGF/Wnt levels starting just after each cell left the tail bud. For these simulations we used the second, more complex, network (Figure 1B), and modelled the FGF/Wnt decay by assuming that *k* linearly decreases in time. According to our results, when *k* decreases below a given threshold, it arrests the segmentation clock oscillations in such a way that well defined groups of PSM cells stop cycling at roughly the same time. Very similar results were obtained when the model was modified to account for additional delays associated to the interactions between the Notch and Wnt pathways. If, as some people suspect, cycling arrest triggers the processes that eventually lead to the creation of a somite, then these results may explain the periodic formation of equally sized somites in mice embryos.

It is important to emphasise that, in order for the PSM cells to stop cycling in a discrete fashion, the delayed negative feedback loop associated with gene N must be capable of generating sustained oscillations by itself. Otherwise, if the feedback loop of gene W oscillates by itself and makes the expression of gene N cycle, the cells along the PSM stop oscillating at almost uniformly distributed times. This result, together with the fact that the W feedback loop must generate sustained oscillations (recall that it still cycles in the absence of *N* oscillations), allows us to assert that the spontaneous oscillation of both pathways is essential to the proper performance of the segmentation clock and its interaction with the determination front. Aulehla and Pourquié [19] posed the question of a possible hierarchy between the Wnt and Notch signalling pathways. In that regard, our results suggest that no such hierarchy exists between these pathways in mice.

The dynamics of the network in Figure 1B not only are compatible with numerous experimental observations regarding somitogenesis in mice, but also provide a possible explanation for the aggregation of PSM cells into blocks and predict the existence of bistability. An important feature of bistability is hysteresis: for a cell to stop oscillating, *k* must decrease below *k*′ (see Figure 3B), but in order to make it cycle again, *k* must surpass a second threshold *k*″*>k*′. This behaviour is a prediction of the model that can, in principle, be tested experimentally to validate the model. Hysteresis can also be biologicaly significant since it would prevent cells in which oscillations have already stoped from cycling again due to fluctuations in the extracellular concentrations of FGF and/or Wnt.

## Supporting Information

Movie S1 Animation of the time evolution of 18 PSM cells after they leave the embryo tail bud. To develop this animations we used the data plotted in the *N vs. t* curves of Figure 4A.
(0.75 MB MOV)

Movie S2 Animation of the time evolution of 18 PSM cells after they leave the embryo tail bud. To develop this animations we used the data plotted in the *N vs. t* curves of Figure 4B.
(0.77 MB MOV)
